# Effects of environmental factors on sensitivity of *Cryptococcus neoformans* to fluconazole and amphotericin B

**DOI:** 10.1093/femsle/fnab040

**Published:** 2021-04-20

**Authors:** Tyler Carlson, Emily Lupinacci, Katie Moseley, Srikripa Chandrasekaran

**Affiliations:** Department of Biology, Furman University, 3300 Poinsett Highway, Townes 171-G, Greenville SC 29613, USA; Department of Biology, Furman University, 3300 Poinsett Highway, Townes 171-G, Greenville SC 29613, USA; Department of Biology, Furman University, 3300 Poinsett Highway, Townes 171-G, Greenville SC 29613, USA; Department of Biology, Furman University, 3300 Poinsett Highway, Townes 171-G, Greenville SC 29613, USA

**Keywords:** Cryptococcus, fungal pathogenesis, drug resistance, antioxidants, reactive oxygen species

## Abstract

*Cryptococcus neoformans* is a leading cause of fungal meningitis in immunocompromized populations. Amphotericin B (AMB) and fluconazole (FLC) are common anticryptococcal agents. AMB treatment leads to severe side-effects. In contrast, FLC-based therapy is relatively safe, although *C. neoformans* often develops resistance to this drug. *C. neoformans* must adapt to the challenging environment of the human host. Environmental effects on potency of AMB and FLC and development of drug resistance remain poorly characterized. Here, the effects of nutrients, temperature and antioxidants on susceptibility of *C. neoformans* towards FLC and AMB were investigated. Limited nutrients led to a decrease and an increase of sensitivity towards FLC and AMB, respectively. Co-treatment with various antioxidants also demonstrated reciprocal effects on susceptibility towards FLC and AMB. In contrast, elevated temperature increased the efficacy of both drugs, although the effect on FLC was more drastic as compared to that of AMB. In addition, temperatures of 37°C and above prevented development of FLC resistance. Our study pointed to a critical role of the environment on susceptibility towards AMB and FLC and revealed reciprocal effects towards these antifungal drugs, reflecting contrasting modes of action of AMB and FLC.

## Teaser

This study defines the effects of nutrient limitation, elevated temperature, and presence of anti-oxidants on susceptibility of a fungal pathogen, *Cryptococcus neoformans*, to fluconazole and amphotericin B.

## INTRODUCTION

A fungal pathogen *Cryptococcus neoformans* causes severe pulmonary infection and meningitis in immunocompromized individuals representing the second-largest cause of HIV-related mortalities (Idnurm *et al*. 2005; Park *et al*. 2009). Although improved access to antiretroviral therapies has caused a significant decrease in cryptococcal infection in recent decades, as of 2014 there were still an estimated 223,000 cases of cryptococcal meningitis globally, and around 180,000 deaths resulting from HIV-associated cryptococcosis (Rajasingham *et al*. 2017; Williamson *et al*. 2017). Antifungal drugs commonly used to treat cryptococcosis are the fungistatic tri-azole drug fluconazole (FLC) and the fungicidal polyene drug, amphotericin B (AMB; Mourad and Perfect 2018).

FLC prevents fungal growth by inhibiting the enzyme lanosterol 14α-demethylase, encoded by *ERG11*, which is responsible for the conversion of lanosterol to ergosterol (Goa and Barradell 1995). Fungal ergosterol is functionally analogous to cholesterol in animals; it is crucial for the proper functionality of the cell membrane (Rodrigues 2018). FLC-resistant fungi commonly exhibit a mutation in the *ERG11* gene, which eliminates inhibition of Erg11 by FLC (Berman and Krysan 2020). Alternatively, FLC-resistant strains possess an increased copy number of *ERG11* resulting from aneuploidy (Berman and Krysan 2020). In *C. neoformans*, the predominant form of FLC resistance, called heteroresistance, is intrinsic and results from formation of aneuploidy leading to overexpression of key resistance genes, including *ERG11* (Mondon *et al*. 1999; Sionov *et al*. 2009; Sionov *et al*. 2010).

AMB belongs to the polyene class of drugs commonly utilized in the treatment of systemic fungal infections (Torrado *et al*. 2008). Unlike FLC, which inhibits fungal growth, AMB kills fungal cells by directly binding to ergosterol in the plasma membrane rather than disrupting the ergosterol biosynthesis pathway (Mesa-Arango, Scorzoni and Zaragoza 2012). AMB bound to ergosterol forms transmembrane channels, which cause cellular ions such as K^+^, Na^+^, Cl^−^ and H^+^, to leak out and ultimately leads to cell death (Gray *et al*. 2012). In contrast to FLC, AMB exhibits significant toxicity to humans (Sabra and Branch 1990).

Both AMB and FLC lead to generation of oxidative stress in fungi due to the production of Reactive Oxygen Species (ROS), although this effect is significantly more pronounced upon treatment with AMB (Mesa-Arango *et al*. 2014; Mahl *et al*. 2015; Guirao-Abad *et al*. 2017; Peng *et al*. 2018; Dbouk *et al*. 2019). ROS such as hydrogen peroxide, oxides, or hydroxide are highly unstable cytotoxic molecules that generate free radicals which can cause molecular and cellular damage (Auten and Davis 2009). Consistent with FLC causing an increase in ROS, treatment with ascorbic acid (AA) restores growth of *C. albicans* and *C. neoformans* from FLC-induced inhibition (Wang *et al*. 2009; Peng *et al*. 2018; Dbouk *et al*. 2019). Interestingly, while treatment of *C. neoformans* with FLC and the antioxidants retinoic acid (RA), ascorbic acid (AA) and pyrrolidine dithiocarbamate (PDTC), can lower the sensitivity of *C. neoformans* to FLC, the antioxidant glutathione (GSH) exhibits little to no rescue from FLC inhibition (Dbouk *et al*. 2019). Whether the efficacy of AMB is altered in the presence of various antioxidants has not been determined.

In addition to oxidative stress, other environmental factors such as elevated temperature and limited nutrient availability may influence the efficiency of antifungal drugs, as also shown recently in *C. neoformans* (Agrawal *et al*. 2007; Coenye *et al*. 2008; Altamirano, Simmons and Kozubowski 2018; Rosenberg *et al*. 2018; Berman and Krysan 2020). *C. neoformans* that acquires resistance to FLC within the human host must simultaneously adapt to multiple stressors (Brown, Campbell and Lodge 2007). Hence it is important to understand the relationship between environmental stressors such as elevated temperature or low nutrient content and susceptibility to antifungal drugs. Although a recent study has demonstrated that sensitivity of *C. neoformans* to FLC increases at higher temperature and under rich media conditions, the underlying mechanisms responsible for these environmental effects remain unclear (Altamirano, Simmons and Kozubowski 2018). In the filamentous fungus, *Fusarium*, the MIC of AMB is minimally reduced at high temperatures (Pujol *et al*. 1997). Whether temperature and/or poor nutrient content affect sensitivity of *C. neoformans* to AMB is not established.

In this study, we compared the effects of temperature and low nutrient conditions on the efficacy of FLC and AMB towards *C. neoformans*. Strikingly, the low nutrient conditions had a reciprocal effect on susceptibility of *C. neoformans* to FLC and AMB, increasing the sensitivity to AMB while decreasing susceptibility to FLC at room temperature, when compared to growth in rich nutrient media. Presence of antioxidants also exhibited reciprocal effects on the cells treated with FLC and AMB. However, elevated temperatures did not exhibit such reciprocal effects on the susceptibility to FLC and AMB, suggesting that the mechanisms behind the effects of low nutrients, antioxidants, and high temperature on susceptibility to these antifungals are not the same. Our study points to a complex nature of the environmental effects on the susceptibility of *C. neoformans* towards FLC and AMB and underlies the differing mode of action of these two antifungals.

## MATERIALS AND METHODS

### Reagents used

Ascorbic acid (AA; Fisher Scientific, Cat No. A61–25, CAS 5081–7, Waltham, MA, USA) was prepared from a stock of 1 M and used at 10 mM. A reduced form of glutathione (GSH; Alfa Aesar, Cat No. AAJ6216606, CAS 70–18–8, Ward Hill, MA, USA) was prepared from a stock of 0.5 M and used at 10 mM. Pyrrolidine dithiocarbamate (PDTC; Cayman Chemicals, Cat No. 20713, CAS 5108–96–3, Ann Arbor, MI, USA) was prepared from a stock of 10 mM and used at 10 μM. Retinoic acid (RA; Cayman Chemical, Cat No. 11017, CAS 302–79–4) was prepared from a stock of 100 mM (dissolved in dimethyl sulfoxide (DMSO)) and used at 1 mM. Fluconazole (FLC; Cayman Chemical, Cat No. 11594, CAS 86386–73–4) was dissolved in DMSO as a 50 mg/mL stock and used at 32 μg/mL. Amphotericin B (AMB; Fisher Scientific, Cat No. BP 928 250) was prepared as a stock of 2 mg/mL (dissolved in dimethyl sulfoxide (DMSO)) and used at 2 μg/mL.

### Strains and media

*C. neoformans* var. grubii wild type (strain H99 Stud) is the derivative of the original strain isolated in 1978 by John Perfect at Duke University (ATCC 208821) that has been passaged through a rabbit. Cells were grown on rich YPD media without amino acids (1% yeast extract, 2% peptone, 2% dextrose and 2% agar, obtained from Fisher Scientific, BD Difco YPD broth, Cat No. DF-0428–07–7, Franklin Lakes, NJ, USA), or nutrient-poor YNB media (ammonium sulfate at 5 g/mL, vitamins, trace elements and salts, obtained from Fisher Scientific, MP biomedicals, yeast nitrogen base with Ammonium Sulfate, Cat No. MP114027512, Santa Ana, CA, USA). Unless specifically stated, cell cultures were routinely maintained at ∼25°C, exposed to atmospheric CO_2_.

### Drug sensitivity spot growth assays

Cells were grown in liquid YPD broth for 16 h. Next, cells were diluted to an Optical Density of OD_600_ = 0.1 and refreshed in YPD liquid media for 4 h and then counted using a Neubauer Hemocytometer. Spot growth assays were performed with a 10-fold serial dilution of cells such that 2 µL contained either 10^4^, 10^3^, 10^2^ or 10 cells and were carefully spotted onto YPD control plates (YPD plates supplemented with DMSO as vehicle control), YPD plus 32 µg/µL FLC, or YPD plus 2 µg/ml AMB and either no antioxidants or 1 mM RA, 10 mM AA, 10 µM PDTC or 10 mM GSH. Plates were incubated at ∼ 25°C for 2–3 days before recording the data.

### Disk diffusion assay

Cells were grown in YPD or YNB liquid broth for 16 h, diluted to an OD_600_ = 0.1 and refreshed for 4 h in YPD or YNB medium. Cell density was counted using a hemocytometer and ∼2 × 10^6^ cells were plated onto YPD (from YPD liquid media) or YNB (from YNB liquid media) semi-solid media plates. 10 min after cell inoculation, 6.6 mm cotton disks were lightly placed on top of the media as to not break the surface. Depending on the experiment, either increasing amounts of 25, 50 and 100 μg of FLC, or increasing amounts of 5, 10 and 25 μg AMB were added to the top end of the disk in order for the FLC or AMB to diffuse throughout the area surrounding the disk. In each case, 20 μL of PBS (with no drug for the control or containing the desired amount of the drug, as indicated above) was added to each disk. The cells were incubated for at least 48 h at ∼25°C prior to data recording. All treatments were done in triplicate. Each zone of inhibition was measured and the results from each of the three replicate experiments were averaged.

### Minimum inhibitory concentration test strip

Cells were grown overnight in liquid YPD or YNB and then refreshed the following morning in the corresponding medium for 4 h. Approximately 2 million cells were plated onto either YPD or YNB agar medium and then spread with a cotton-tipped applicator. 15 min after cell inoculation, an MIC test strip, which contained a gradient of either FLC (Fisher Scientific, Liofilchem FLC 0.016–256 μg/mL, Cat No. 22–777–964) or AMB (Fisher Scientific, Liofilchem, AMB, 0.002–32 μg/mL) was carefully added to the agar surface. The plates were incubated for 72 h prior to data recording; the MIC was read where the edge of the zone of inhibition intersects the test strip. In order to determine the effect of temperature, the plates with the MIC strips were placed at incubators set at 25, 30, 32, 35, 37 and 39°C.

### Statistical analysis

For the analysis of the data from the disk diffusion assay, the Shapiro–Wilk Test was utilized to test for normality and the Bartlett Test was employed to test for equality of variance. Since both conditions were met, a one-way ANOVA was performed to test for the significance in difference between means.

## RESULTS AND DISCUSSION

### The effect of nutrient content on sensitivity to FLC and AMB

**Figure 1.**
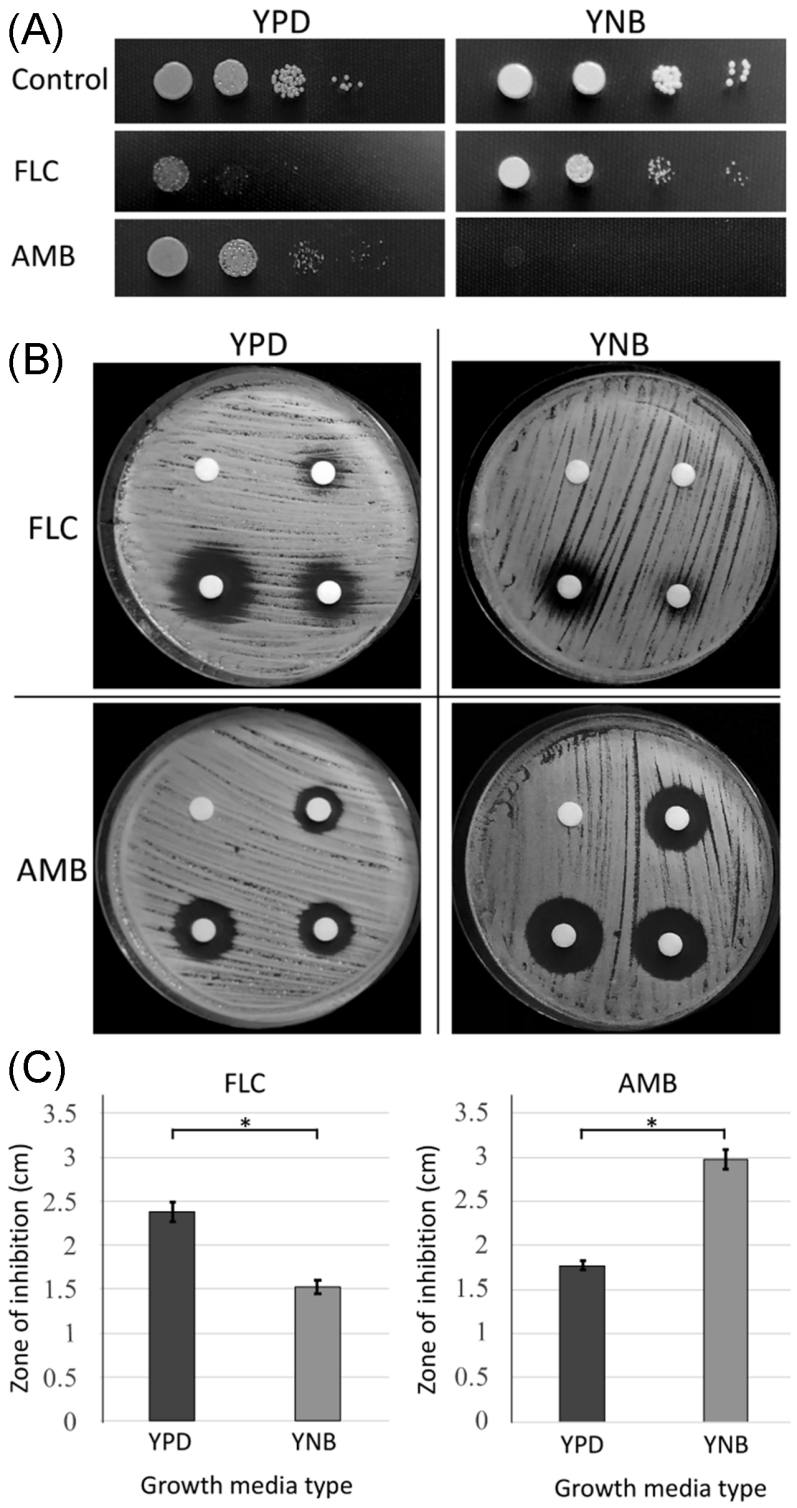
The effect of nutrient limitation on susceptibility towards FLC and AMB **A**. A spot growth assay, in which cells were incubated for 72 h at room temperature (∼ 24–28°C) on either rich YPD or nutrient poor YNB semi-solid media, with no drugs or media supplemented with 32 μg/mL FLC, or 2 μg/mL AMB. **B**. Disk diffusion assay was performed with cells spread on either YPD or YNB media. The disks contained either FLC (0, 25, 50 and 100 μg, clockwise) or AMB (0, 5, 10 and 25 μg, clockwise). **C**. The graphs represent the comparison of the average zone of inhibition caused by FLC (100 μg-containing disk) and AMB (25 μg-containing disk) with included error bars based on standard deviation, based on three replicates, as exemplified in (B). Stars indicate statistical significance (*P* < 0.001).

It has been demonstrated previously that FLC causes more growth inhibition when the culture of *C. neoformans* is maintained on rich YPD media as compared to the nutrient-poor YNB media (Altamirano, Simmons and Kozubowski 2018). Specifically, lower percentage of *C. neoformans* cells developed into visible colonies on YPD semi-solid media as compared to YNB media, both supplemented with 32 μg/mL FLC (Altamirano, Simmons and Kozubowski 2018). What could account for this difference in susceptibility towards FLC? The growth rate of *C. neoformans* is slower in the nutrient poor YNB media as compared to the rich YPD media (Altamirano, Simmons and Kozubowski 2018). Previous studies have demonstrated a nearly linear relationship between growth rate of *Saccharomyces cerevisiae* and the ergosterol content. Specifically, the higher growth rate was associated with a decrease of the levels of ergosterol (Arnezeder and Hampel 1990). As the inhibitory effect of FLC is based on the depletion of ergosterol, better survival on YNB media may be associated with elevated content of the ergosterol when cells are growing relatively slower under these poor nutrient conditions. If this were the case and considering that AMB killing mechanism depends on ergosterol binding (Young, Hull and Heitman 2003), *C. neoformans* should exhibit higher susceptibility to AMB on YNB as compared to YPD media. To test this possibility, we spotted 10-fold serial dilutions of the overnight *C. neoformans* cultures on either semi-solid YPD or YNB medium, either containing no antifungal drugs as control, 32 μg/mL FLC, or 2 μg/mL AMB. Consistent with previously published results, we found that *C. neoformans* cells exposed to FLC formed more colonies on YNB media compared to YPD media, when cells were grown at 25°C (Altamirano, Simmons and Kozubowski 2018). Interestingly, *C. neoformans* cells exposed to AMB were more affected when grown on YNB compared to YPD media (Fig. 1A). This result was consistent with the hypothesis that the poor nutrient media promote better survival of *C. neoformans* in the presence of FLC due to a relatively higher ergosterol content under those growth conditions. To corroborate those findings, we carried out an experiment in which cells were exposed to a drug concentration gradient in a disk diffusion assay. Zones of growth inhibition around FLC-containing disks were larger on YPD media as compared to YNB media, confirming that *C. neoformans* cells are more sensitive to FLC in YPD medium as compared to YNB. In contrast, the zones of inhibition around AMB-containing disks were larger in YNB media than in YPD media, indicating that *C. neoformans* cells are more sensitive to AMB in YNB medium as compared to YPD. The differences between the zones of inhibition (between FLC disks in YPD v/s YNB and between AMB disks in YPD v/s YNB) were both significant (*P* value < 0.001; Fig. 1B and C). These results indicate a reciprocal effect of nutrient content on the susceptibilities of *C. neoformans* towards FLC and AMB. While former studies based on *S. cerevisiae* (Arnezeder and Hampel 1990) suggest that our results may stem from differences in ergosterol levels when cells are grown under low and high nutrient conditions, future studies will be needed to directly test ergosterol content in *C. neoformans* grown on various media representing rich and poor nutrient content.

### The effect of temperature on sensitivity to FLC and AMB

**Figure 2.**
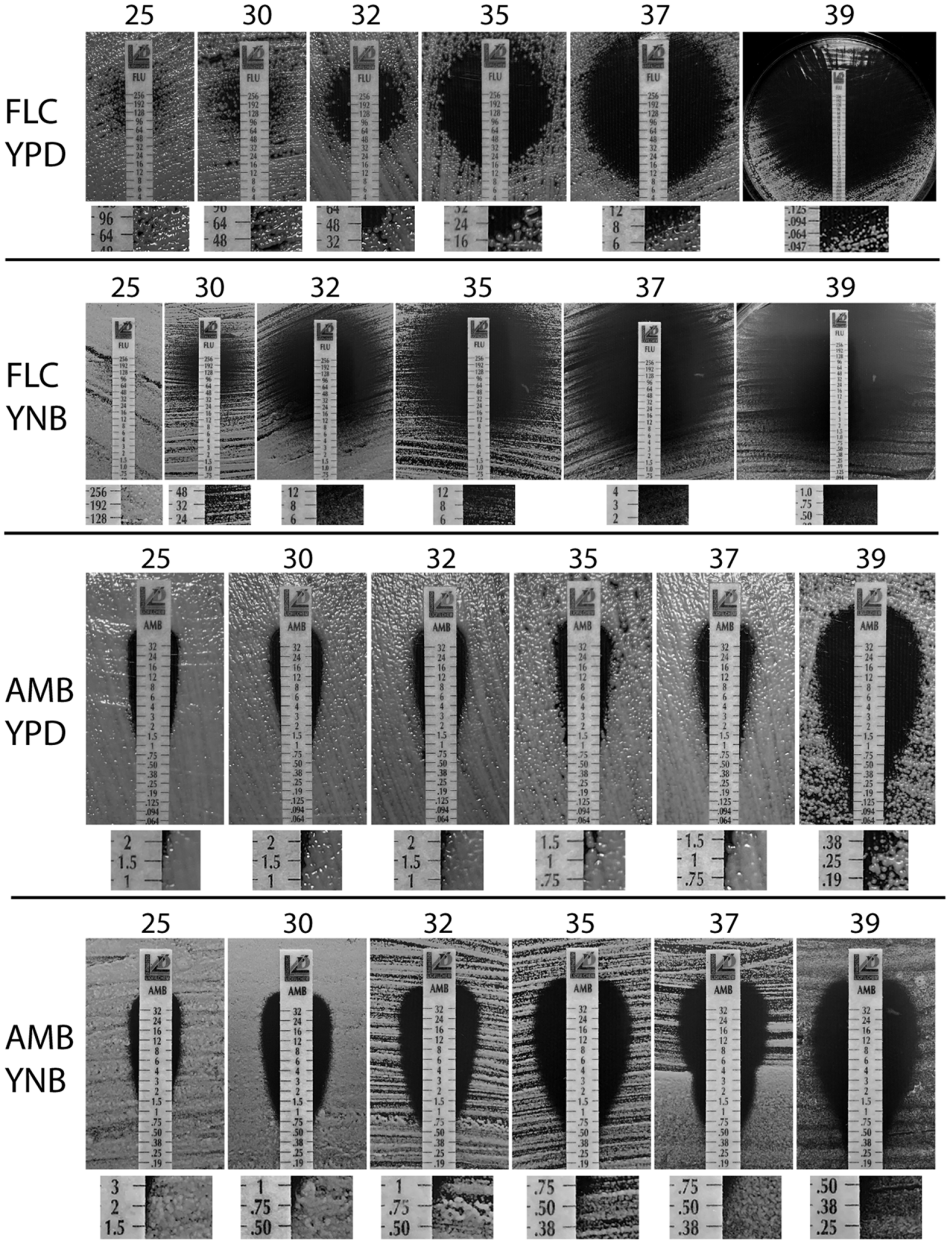
The effect of temperature on susceptibility towards FLC and AMB. Cells were spread on either YPD or YNB semi-solid media and strips containing concentration gradient of either FLC or AMB were applied to each plate. Plates were imaged after 72 h of incubation at various temperatures (°C), as indicated on top of each panel. Below each panel, a magnified area corresponding to the approximate MIC values is shown.

The reciprocal results between effects of rich versus nutrient poor media for FLC and AMB were observed at room temperature (∼ 25–28°C). We wished to further explore the effect of nutrient content at increasing temperatures in a temperature-controlled environment. Temperature of the human host (∼37°C) was particularly relevant in this case. Previous studies have indicated that an increase of temperature from 25 to 30°C results in a decrease in number of *C. neoformans* colonies when incubated on media supplemented with 32 μg/mL FLC (Altamirano, Simmons and Kozubowski 2018). Temperatures above 35°C led to reduction of heteroresistant *C. neoformans* colonies on FLC media (Mondon *et al*. 1999). Hypothetically, this phenomenon may be also associated with similar mechanism responsible for reciprocal effects of nutrient content on susceptibility towards FLC and AMB. If this were the case, we would expect to observe reciprocal effect of temperature on susceptibility to AMB. To test the effect of temperature on susceptibility to FLC and AMB when cells are grown on YPD and YNB media, we performed the MIC strip assay (Fig. 2). In this assay, standardized strips are utilized that contain increasing concentration of the drug along a scale which allows a readout of the minimum inhibitory concentration (MIC) values, if the test is performed under specified standard conditions. Although in this case it was necessary to deviate from standard conditions (vary the media type and the temperature), utilization of this assay has allowed a more quantitative assessment. For the purpose of comparison, we further call the values read from the strips as the MIC values, even though these values do not refer to the standard MIC. Cells were plated on either YPD or YNB media and incubated with the strips of FLC and AMB at 25, 30, 32, 35, 37 or 39°C for 72 h prior to imaging. At 25°C, there was no visible effect of FLC on growth of *C. neoformans* when incubated on YNB medium, but when the culture was maintained on YPD, the inhibition of *C. neoformans* at 25°C was evident (Fig. 2). On both YPD and YNB media, the zones of growth inhibition caused by FLC increased significantly upon exposure of *C. neoformans* to increasing temperatures (Fig. 2). While on YPD there was a clear boundary between growth and no growth zones, on YNB media, the boundary between growth and no growth zones was blurred as it contained a relatively wide transition area consisting of colonies of gradually decreasing sizes (Fig. 2). This has hampered an accurate reading of the MIC values. Accordingly, at temperatures equal to or higher than 32°C, MIC values for FLC in YNB media appeared relatively lower than the MIC for FLC in YPD media. This would suggest that while at room temperature *C. neoformans* was more sensitive to FLC in YPD media compared to YNB media, at higher temperatures (32, 35, 37 and 39°C) it was more sensitive to FLC in YNB media as compared to YPD media. However, the aforementioned blurred zone of inhibition on YNB media prevents us from drawing such a conclusion.

At 25°C, there was no clear difference in susceptibility to AMB between YPD and YNB, in contrast to spot growth assay or the disk diffusion assay maintained at room temperature (Fig. 2 compared to Fig. 1). In contrast to FLC, the boundary between growth and no growth zones for AMB on YNB media was relatively well defined, which has allowed for the comparison of the MIC values. At increasing temperatures, estimated MIC of AMB was decreased when *C. neoformans* was maintained on either YPD or YNB media (Fig. 2), although this effect was more pronounced when cells were maintained on YNB media as compared to YPD. Consistently, the MIC of AMB was lower for YNB when compared to YPD at corresponding growth temperatures, suggesting that at higher temperatures, growth in poor nutrient conditions led to a more significant inhibition of *C. neoformans* compared to rich YPD media. In addition, the effect of temperature was significantly less pronounced for media supplemented with AMB as compared to media supplemented with FLC. This was particularly striking at 39°C. Collectively our data demonstrate that temperature potentiates inhibitory effects of both FLC and AMB. However, the effect of temperature is more pronounced for FLC. Thus, in contrast to the effect of the nutrient availability, an increase in temperature does not lead to reciprocal results for FLC and AMB.

### The effect of antioxidants on sensitivity to FLC and AMB

**Figure 3.**
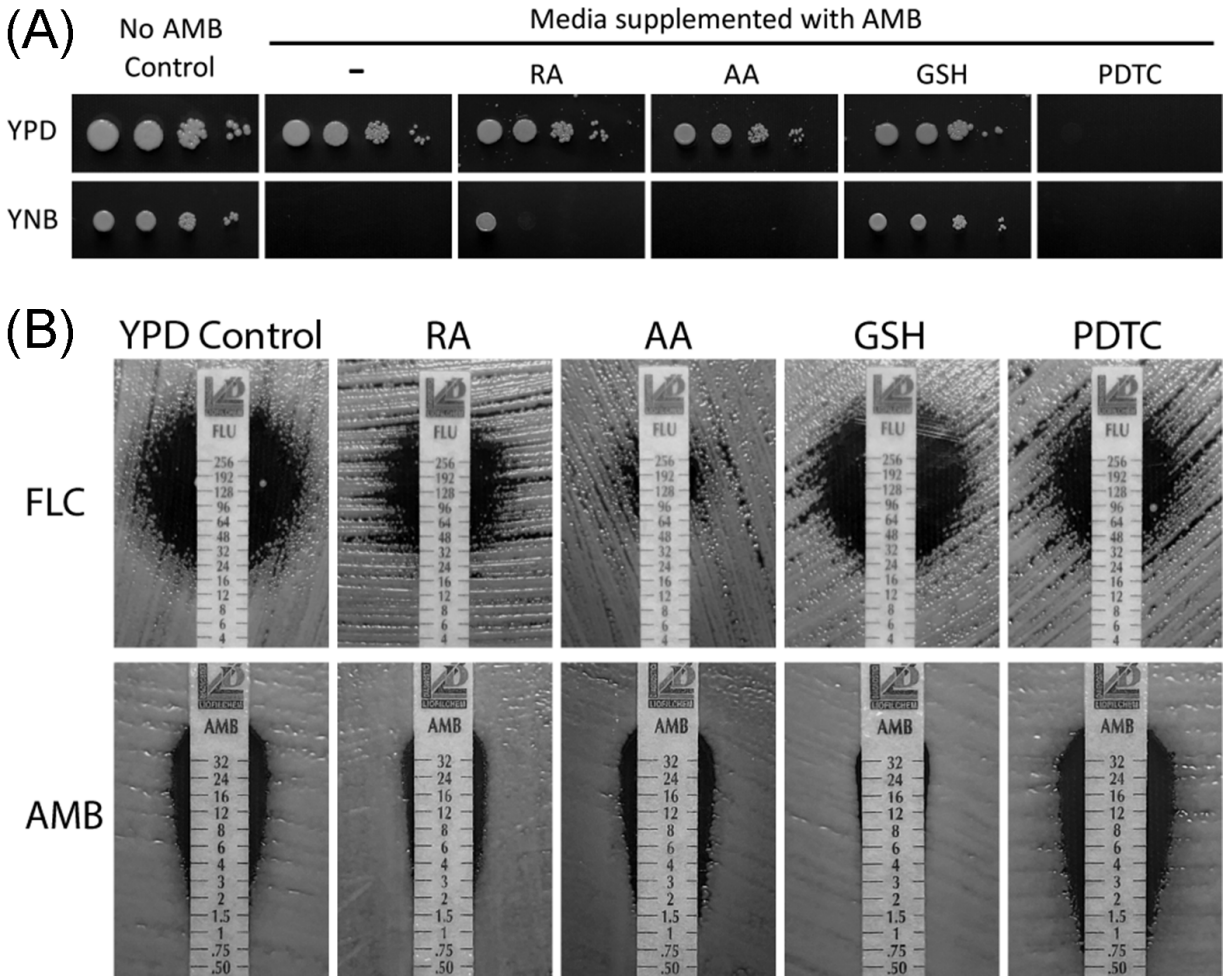
The effects of antioxidants on susceptibility towards FLC and AMB **A**. A spot growth assay on YPD or YNB control media or media supplemented with 2 μg/mL AMB without an antioxidant or with one of the antioxidants as indicated. **B**. The effect of the antioxidants on susceptibility towards FLC and AMB was tested. Cells were incubated at 35°C on YPD semi-solid media with the strips containing a concentration gradient of the antifungal compounds, as indicated.

One factor that could potentially contribute to the influence of nutrient content and/or the temperature on drug susceptibilities is the ROS. For instance, growth of *C. neoformans* in the presence of L-Dopa leads to an increase of melanization and a decrease in susceptibility to AMB (Wang and Casadevall 1994). AMB causes a dramatic increase of ROS in *C. neoformans*, which likely contributes to the killing effect of this drug (Sangalli-Leite *et al*. 2011; Mesa-Arango *et al*. 2014). FLC also leads to an increase of ROS, although the levels of ROS resulting from FLC treatment seem significantly lower as compared to those stimulated by AMB (Peng *et al*. 2018; Dbouk *et al*. 2019). It has been shown that co-treatment of *C. neoformans* with FLC and the antioxidants, retinoic acid (RA), ascorbic acid (AA) and pyrrolidine dithiocarbamate (PDTC) prevents the increase of ROS and has a significant effect by lowering the susceptibility towards FLC (Dbouk *et al*. 2019). Surprisingly, the effect of yet another classic antioxidant, glutathione (GSH) had a minimal effect on susceptibility towards FLC (Dbouk *et al*. 2019). Given the reciprocal effects of the nutrient and differences in the effect of temperature between FLC and AMB, we wished to probe the effects of the antioxidants on the susceptibility towards these two drugs. Strikingly, the only antioxidant that led to improved growth in the presence of AMB was GSH, while RA had only a mild growth rescue effect (Fig. 3A). Interestingly, PDTC potentiated the inhibitory effect of AMB as no growth was detected on YPD media containing 2 μg/mL of AMB and PDTC (Fig. 3A). To assure more quantitative outcome of the experiments, we utilized the MIC strips when cells were grown at 35°C (Fig. 3B). Consistent with previous results, the addition of antioxidants RA, AA and PDTC but not GSH led to an increase of the MIC of FLC (Fig. 3B). In contrast, the MIC of AMB was visibly reduced only in the presence of GSH but not in the presence of RA, AA or PDTC (Fig. 3B). Consistent with the outcome of the spot growth assay (Fig. 3A), the zone of growth inhibition was larger for cells exposed to both AMB and PDTC, as compared to those exposed to AMB alone (Fig. 3B).

What could account for the differences in the effects of antioxidants on the inhibitory/killing potential of FLC and AMB? One possible explanation is that while the inhibitory effect of AMB is highly influenced by ROS, the contribution of ROS to FLC-mediated inhibition is relatively less significant. Accordingly, the reversal of inhibitory effects of FLC by AA, RA and PDTC may not be due to antioxidant potential of these compounds. Van Hauwenhuyse *et al*. have demonstrated that the growth rescue by AA of the FLC treated *C. albicans* was due to an increase of expression of Upc2, a transcriptional regulator of genes involved in ergosterol biosynthesis (Van Hauwenhuyse, Fiori and Van Dijck 2014; Yang *et al*. 2015). A study evaluating the effects of these antioxidant compounds on gene expression in *C. neoformans* should examine this possibility. Alternatively, our results may stem from the fact that different species of ROS are primarily generated in the presence of AMB and FLC. In this case, types of antioxidants acting against specific ROS species may reverse the inhibitory effects of these antifungal drugs. For instance, reduced GSH is primarily responsible for neutralizing peroxide radicals (Nimse and Pal 2015), so potentially AMB is responsible for generating high levels of peroxide radicals, in contrast to FLC. Future studies should differentiate between these possibilities.

### The effects of nutrient content and temperature on the development of resistance to FLC

Development of *C. neoformans* resistance to FLC constitutes a considerable challenge for the anticryptococcal therapy (Bongomin *et al*. 2018). Heteroresistance is the most common type of FLC resistance in *C. neoformans*, where aneuploid cells with additional copies of *ERG11* and/or genes encoding drug pumps are selected under drug selective pressure (Sionov *et al*. 2009; Sionov, Chang and Kwon-Chung 2013). Whether aneuploidy can be stimulated by FLC or can be preexisting in the population remains unclear (Altamirano *et al*. 2017; Chang, Khanal Lamichhane and Kwon-Chung 2018). We noted that while cells treated with FLC developed resistant colonies within the growth inhibition zone on YPD media at temperatures up to 35°C, such colonies were rarely observed on YNB media (Fig. 2). No clear resistance to AMB within the inhibitory zone was observed under tested conditions. This is consistent with the fact that FLC is fungistatic whereas AMB is fungicidal. The prohibitive effect of temperature on the development of FLC resistant colonies on YPD media puts into question the relevance of such *in vitro* studies to human infection as the development of FLC resistance has been documented in the murine model of infection and constitutes a challenge during anticryptococcal treatments (Sionov, Chang and Kwon-Chung 2013). Nonetheless, clearly temperatures above 35°C cause an increase in the effectiveness of FLC treatment *in vitro* and contribute to eliminating FLC resistance when cells are incubated on rich YPD media. While the development of the resistance of *C. neoformans* to FLC has been documented in the clinical settings (Smith *et al*. 2015; Mpoza, Rhein and Abassi 2018; Stone *et al*. 2019), our data suggest that the temperature of the host potentiates effectiveness of FLC and counteracts the development of resistance *in vitro*. While this seems contradictory, it underscores the complexity of the host environment. Recently, the dynamics of the aneuploidy driven FLC resistance of *C. neoformans* has been investigated and indicated a critical role of combined therapy with the addition of a fungicidal drug, flucytosine (Stone *et al*. 2019). Future studies should further aid in our understanding of the dynamics of the development of antifungal resistance during the infection.

The effect of poor nutrient content on eliminating FLC resistant colonies is intriguing. One possibility to explain this phenomenon is the relatively slow growth rate under nutrient limitation. Under such conditions, cells may have sufficient time to resolve potential DNA and chromosomal instability based on active cell cycle checkpoints. This would in turn reduce a chance for the development of aneuploidy that is known to fuel the formation of resistant colonies. Another possibility is that growth in rich media leads to more ROS production, which may lead to chromosomal instability and/or increase mutation rate stimulating aneuploidy. However, our data suggest that application of antioxidants does not eliminate the development of resistant colonies within the zone of inhibition, which makes this hypothesis unlikely (Fig. 3). An analysis of gene expression under rich and poor nutrient conditions in the presence of FLC could potentially reveal key genes and pathways associated with the development of resistance. For instance, the expression of the gene encoding App1 (antiphagocytic protein 1), a known virulence factor of *C. neoformans*, is affected under low nutrient environment, elevated upon FLC treatment, and influences susceptibility to FLC (Ghaffar, Orr and Webb 2019).

Under experimental conditions at which FLC resistance has occurred, the colonies observed within the zone of inhibition likely constitute aneuploids developed based on the intrinsic heteroresistance of *C. neoformans* (Sionov *et al*. 2010). However, we cannot exclude a possibility that under some conditions tested here, drug resistant colonies result from mutations in key resistance genes, including *ERG11*. Future studies will address this possibility.

## CONCLUSION

We find that at temperature ∼25°C, *C. neoformans* survives better when exposed to FLC on YNB media compared to rich YPD media and the effect of media type is the opposite when cells are treated with AMB. The effect of temperature does not exhibit similar reciprocity, as both FLC and AMB-treated cells are more affected by the drugs at higher temperatures. Cells treated with FLC develop into drug resistant colonies more readily when incubated on YPD media as compared to YNB and when the temperature is at or below 35°C. Finally, of the four antioxidants (RA, AA, GSH and PDTC), GSH is the only one to have a clear rescuing effect on AMB-treated cells while having no such effect on FLC-treated cultures. Thus, some of the environmental factors exhibit opposite effects on FLC and AMB-mediated inhibition of *C. neoformans*, which should be considered when implementing anticryptococcal therapies.
